# Retrospective Mid-Term Follow-Up of Posttraumatic and Iatrogenic Neurovascular Complications in Surgically Treated Paediatric Patients with Distal Humerus Fracture

**DOI:** 10.3390/children9091349

**Published:** 2022-09-04

**Authors:** Alexander Gutwerk, Peter Behrendt, Svenja Vetter, Leif Menzdorf, Edward Oates, Sebastian Jazra, Sebastian Lippross, Tim Klüter, Andreas Seekamp, Matthias Weuster

**Affiliations:** 1 Department of Orthopedic, Trauma, Hand and Reconstructive Surgery, DIAKO Clinic, 24939 Flensburg, Germany; 2 Department of Trauma Surgery, Orthopedics and Sportsorthopedics, Asklepios St. Georg, 20099 Hamburg, Germany; 3 Department of Anatomy, Christian-Albrechts-University, 24118 Kiel, Germany; 4 Department of Orthopedics and Traumatology, University Medical Center Schleswig-Holstein, Campus Kiel, 24105 Kiel, Germany

**Keywords:** paediatric elbow, supracondylar, epicondylar, fracture, outcome, DASH score

## Abstract

Background: The purpose of the study was to investigate and describe neurovascular complications and mid-term clinical outcomes of operatively managed fractures of the distal humerus in a paediatric population. Neurovascular injuries are common in these fractures, but reports about their implications for mid-term clinical outcomes is sparse. Methods: A single-centre retrospective study was conducted at a university teaching hospital investigating paediatric patients who underwent operative management of a distal humerus fracture between 2014 and 2018. Patient demographics, fracture classification, pre-, peri- and postoperative neurovascular complications were investigated. Mid-term follow up clinical examination and functional scoring using QuickDASH, the Broberg and Morrey Score (BMS), the Mayo Elbow Performance Score (MEPS) and the Numeric Rating Scale were performed. Results: A total of 84 patients were identified, of which 34 met the inclusion criteria and were available for follow-up clinical examination. The average time to follow-up was 150 weeks (1049.44 days ± 448.54). Ten primary traumatic neurovascular complications were identified, the majority of which involved the median nerve. Primary traumatic dissection of the brachial artery was recorded in three patients. Secondary iatrogenic nerve injury was documented in five patients after previously normal clinical examination. At follow-up, the average QuickDASH score was 3.0 ± 4.3, BMS was 98.6 ± 3.4 and MEPS was 97.1 ± 3.3 points. Conclusions: The mid-term clinical outcome following surgical management of distal humerus fractures is excellent. There is, however, a considerable frequency of both primary and secondary neurovascular complications, which must be considered when opting to treat these injuries surgically.

## 1. Introduction

Following distal forearm fractures, distal humerus fractures are the second most common type of fracture in the paediatric population [1]. Supracondylar fracture is the most frequent type of distal humerus fracture, representing around two-thirds of all patients. This is followed by lateral condyle fractures and medial epicondyle fractures [1,2]. The most common mechanism of injury is a fall onto an outstretched arm. Such falls typically occur on playgrounds, in parks or at home [1,3]. Allowing for seasonal fluctuation, there is an age-dependent distribution of injury: (1) lateral condyle fractures occur most frequently in children around four years of age; (2) the incidence of supracondylar fracture peaks around six years of age; and (3) fractures of the medial epicondyle apophysis reach peak incidence around 11 years of age [4,5].

Complications associated with fractures of the distal humerus are well reported, often differentiated by either fracture classification or operative technique. Primary traumatic and secondary iatrogenic lesions of neurovascular structures remain one of the most significant complications in surgically treated supracondylar fractures [6]. Primary post traumatic nerve injury is reported in around 5% and vascular injury in around 0.7% of reported patients. The radial and median nerve are most frequently involved. 

Surgical treatment is common for these fracture types, reflective of the limited capability of spontaneous deformity correction, especially in the coronal plane [5]. A considerable frequency of surgery-related complications has been reported for different operative techniques, including neurovascular injuries [7,8]. Residual varus or valgus deformity in the coronal plane is reported in around 10% of patients, likely reflecting inadequate intra-operative fracture reduction [4,6]. Similarly, lateral condyle fractures are frequently associated with pseudoarthrosis, leading to cubitus valgus clinical deformity [5,9]. 

Given the frequency of paediatric distal humerus fracture, its treatment is frequently discussed in the paediatric trauma literature regarding timing the type of treatment, the mode of fixation, the duration of immobilisation and the rate of complications [8]. 

Accordingly, the aim of this study was to perform a qualitative analysis of the frequency of both primary traumatic and secondary iatrogenic neurovascular complications along with mid-term clinical outcomes in patients presenting to our facility with distal humerus fractures. 

## 2. Materials and Methods

Study population: This was a retrospective study of children under the age of 17 years who sustained a fracture of the distal humerus. The study was approved by the local ethics committee (number D538/18, November 2018). All surgically treated paediatric patients suffering fractures classified as ICD 10 S42.40–S42.49, managed between 2014 and 2018 in the Department of Orthopaedics and Trauma Surgery at the university hospital of Schleswig Holstein, Campus Kiel (UK-SH, CK) were included. The inclusion criteria were indication for surgery, which was based on the patient’s age and degree of displacement as well as open fractures, displaced type 2 supracondylar humerus fracture (SCHF), type III SCHF, flexion type SCHF and those with collapse of the medial column. 

The exclusion criteria included age greater than 17 years, fractures without surgical intervention (von Laer type I SCHF, acceptable displaced von Laer type II SCHF and transcondylar humerus fracture (TCHF; age dependent), non-displaced epicondylar humerus fracture (ECHF), polytraumatised children and patients who suffered traumatic brain injury. All patients and their parents were offered the opportunity to participate in the follow-up clinical examination. The minimum clinical follow-up duration was fixed to be at least one year. 

Fractures were classified as SCHF, ECHF and TCHF according to radiological assessment of anterior-posterior and lateral plain film radiographs. The ‘von Laer’ classification was used to classify SCHF: Type I—undisplaced fracture; Type II—displaced fracture in one plane; Type III—displacement in two planes; and Type IV—displacement in three planes or complete displacement without osseus contact [5].

Study plan: Medical records were reviewed and assessed for clinical parameters, including age and sex, with further subclassification according to the type of distal humerus fracture. These subgroups were then further reviewed for comorbidities, neurovascular complications and operative techniques. 

The operative techniques included closed reduction and internal fixation with K-wires (CRIF), open reduction and internal fixation with K-wires and open reduction and internal fixation with screw and/or plate fixation (ORIF). 

Documentation of the pre- and postoperative neurovascular status was compiled, including abnormalities in the peripheral pulse, lesions of the brachial artery and sensorimotoric neurological deficit due to injury of the ulna, radial or median nerve.

All patients available for clinical follow-up submitted a standardised questionnaire and underwent clinical examination. Range of motion of the elbow, objective muscle atrophy and strength compared to the contralateral uninjured arm were assessed. The stability of the elbow was evaluated with a varus–valgus stress test. 

We used four different scores to measure the outcome: (1) a shortened version of the Disability of Arm, Shoulder and Hand Score (QuickDASH); (2) the Broberg and Morrey Score (BMS); (3) the Mayo Elbow Performance Score (MEPS); and (4) the Numeric Rating Scale (NRS) for pain.

The QuickDASH is a validated 11-item questionnaire that evaluates symptoms and physical function in patients with upper extremity musculoskeletal disorders [10]. Scores range from 0 to 100 points, with a higher score indicating a higher level of disability. The BMS is an 100-point elbow-specific score consisting of four categories: pain (35 points), motion (40 points), strength (20 points) and stability (5 points) [10]. Scores ≥ 95 points indicate an excellent outcome. As a physician-based score, MEPS assesses the function of the elbow based on pain (with a maximum score of 45 points), ulnohumeral motion (20 points), stability (10 points) and the ability to perform five daily activity tasks (25 points) [10]. The total score ranges from 5 to 100 points. Scores between 90 to 100 points indicate an excellent outcome. In addition, we used the NRS to evaluate pain at ease and under stress. A score of 0 points indicates no pain, while a score of 10 indicates maximum pain. For comparison of the mean score results, patients were divided into three groups based on the age at follow-up (1–7 years, 8–12 years, 13–17 years).

Statistical analysis: A retrospective analysis was performed on data collected from patient records. The results are provided as mean ± standard deviation (SD). Statistical analysis was performed using IBM, SPSS Statistics version 21. Fisher’s exact and Chi-square test were applied. A *p*-value ≤ 0.05 was considered statistically significant.

## 3. Results

After obtaining informed consent, 84 patients out of 92 were included (main cohort). 

Two patients were excluded due to incorrect ICD 10 codes, and two cases of children surgically treated in other hospitals were excluded. Forty-four patients agreed to the clinical examination. Finally, 34 patients attended this examination and were included (follow-up cohort).

In one patient, both arms were fractured, and they were analysed separately. The mean age was 7.8 years, ranging from 1 to 15 years (SD 3.83 years). The age groups were from 1 to 7 years (43 patients), 8 to 12 years (27 patients) and 13 to 15 years (14 patients). The majority of the patients were female: 32 male patients (38.1%), 52 females (61.9%). Of the fractures, 46 affected the left arm, and 38 affected the right arm (54.8%/45.2%). There was one case of a medially open SCHF.

### 3.1. Fracture Classification and Surgical Technique

The most frequently occurring fracture, representing approximately two-thirds of fractures, was supracondylar fracture (n = 55; 12 type 2, 23 type 3 and 19 type 4), followed by fractures of the medial epicondyle (n = 16) and lateral epicondyle (n = 9). Of the remaining fractures, two were transcondylar, one was a combined supracondylar fracture and fracture of the medial epicondyle and one was not suitable for classification under our methodology and was therefore classified as 42.49 (‘other’). 

Significant age-dependent differences within the groups were detected: fractures of the medial epicondyle were the dominant type at the age of 13–15 (42.9%), while supracondylar fractures were dominant in the other age groups (1–7: 81.4%; 8–12: 63.0%). The mean age in the subgroups was 6.6 ± 2.9 for supracondylar fractures, 7.7 ± 4.9 for fractures of the lateral epicondyle and 11.3 ± 2.5 for fractures of the medial epicondyle.

All patients included in the study underwent operative management. The operational procedure depended on the type of fracture (Table 1). Supracondylar fractures were treated with K-wire fixation after either closed (CRIF 69.1%) or open reduction (ORIF 30.9%). The use of two crossed K-wires with radial and ulnar entry points was the most common operative technique (71.2%). Three or four K-wires were used in 21.2% and 5.7% of the patients, respectively. Only one supracondylar fracture was stabilised with a single radial wire. Epicondylar fractures (medial and lateral) were reduced exclusively open (ORIF) and stabilised using a K-wire alone or screws with or without a K-wire (Table 1). The mean stay in the hospital was 3.1 ± 1.3 days for SHCF, 3.4 ± 1.7 days for ECHF and 5.5 ± 0.7 days for TCHF.

### 3.2. Early Perioperative Complications

Ten primary neurovascular complications were identified at the initial clinical examination (Table 2). Nine patients had persisting neurological symptomatology at the time of discharge. Three patients developed bony healing complications, including secondary displacement, malreduction and osteonecrosis. Data available at the six-week follow-up demonstrated a reduction in the range of motion (ROM) of the elbow in 19 patients. One patient with bilateral ECHF presented with physiological ROM. Five patients with ongoing neurological dysfunction were observed. One patient had undergone neurolysis of the ulnar nerve within the first six weeks. No patient with infection, compartment syndrome or Volkmann contracture was documented. Injury to the brachial artery was identified in three patients with a supracondylar fracture. All three were identified as a primary injury at the initial clinical examination. No intra- or postoperative vascular complications were documented. Median nerve dysfunction was the most frequently documented neurological deficit (9 patients).

A detailed summary of both primary traumatic and postoperative neurovascular complications and their association with fracture subtype is presented in Table 3. Five patients with neurological dysfunction were documented postoperatively after normal primary clinical examination. No statistical association was found between posttraumatic vascular injury to the brachial artery and age or fracture subtype. There was no incidence of postoperative vascular complication. Posttraumatic nerve injury was statistically significantly related to age (*p* = 0.009) but not fracture subtype (*p* = 0.905). The incidence of postoperative nerve dysfunction was not associated with the type of operative procedure (*p* = 0.601).

### 3.3. Mid-Term Clinical Follow-Up

A total of 34 patients were available for clinical follow-up examination at a mean follow-up time of 150 weeks (1049.44 days ± 448.54 days) after trauma (Table 4). The mean QuickDASH score was 3.0 ± 4.3, while the mean Broberg & Morrey score was 98.6 ± 3.4. The mean Mayo Elbow Performance score was 97.1 ± 3.3 points. NRS at rest and during movement were assessed. At rest, no patient (0/34) had pain. During movement, the mean NRS was 0.8 ± 1.8. No statistically significant difference (*p* < 0.05 using fisher exact test) was found for scores and NRS in patients with or without neurovascular complications. NRS during movement was not associated with joint stability (Fisher exact test; *p* = 0.727). Additionally, stability of the elbow joint was assessed at follow-up. The authors found only 16 patients’ joints to be stable, whereas 18 had minor valgus instability under 10 degrees. There was no significant difference attributable to fracture subtype. The association between joint stability/instability and QuickDASH was close to statistical significance (Fisher exact test; *p* = 0.069), but surprisingly patients with stable joints had higher QuickDASH scores.

Symptomatic neurovascular dysfunction was initially identified in eight patients in the mid-term follow-up group. Primary traumatic neurovascular lesions accounted for four of these patients: (1) dissection of the brachial artery with dysfunction of the median and radial nerve, (2) isolated dysfunction of the median nerve, (3) isolated dysfunction of the ulnar nerve and (4) dissection of the brachial artery without neurological symptomatology. Secondary/iatrogenic neurological dysfunction accounted for a further three patients: (1) one case of median nerve dysfunction, (2) one radial nerve dysfunction and (3) one case of ulnar nerve dysfunction. One further case of symptomatic ulnar nerve dysfunction was documented at the six-week follow-up and was grouped with the three other secondary complications for the purpose of this analysis.

Residual neurovascular symptomatology was demonstrable in only two of the eight patients at the time of mid-term clinical follow-up (one of which had undergone decompression neurolysis of the ulnar nerve in the initial postoperative period). In the single case of complex primary traumatic dissection of the brachial artery with median and radial nerve dysfunction, a residual neurapraxia with paradoxical temperature sensation was present. In one case of primary traumatic median nerve dysfunction, mild residual tingling paraesthesia was identified together with a loss of extension ROM at the elbow joint and qualitative loss of elbow flexion power. None of the secondary/iatrogenic neurological injuries were evident at the mid-term follow-up.

## 4. Discussion

The incidence of secondary iatrogenic neurological complications was high in our study population, representing around one-third of all identified neurovascular injuries. Involvement of the median nerve was the most frequently observed complication, followed by the radial nerve and the ulnar nerve. Three patients with primary traumatic brachial artery dissection were reported—a previously described but perhaps underdiagnosed complication, with a varying frequency in the literature from 0.7% up to 10% [4,11]. All patients with brachial artery injury occurred in patients with supracondylar fractures. This fracture subtype is classically associated with median nerve and/or brachial artery injury, which represents the most frequently occurring combination [8,11]. Clinical assessment at mid-term follow-up demonstrated mostly excellent results according to all scoring systems, indicating that most complications are clinically transient.

Neurovascular injury in paediatric distal humerus fractures has been reported previously [8,11]. In one large German multicentre study of nearly 900 supracondylar fractures, a primary nerve injury was reported in around 5% of patients (N. radialis 44.4%; N. medianus 35.6%; N. ulnaris 20.0%). Differences between these numbers and those reported in the current study may be attributable to selection bias—the interest in the current studs was the operatively managed fracture population. The indication to operative management is directly related to fracture displacement, which has an inherently higher risk of neurovascular injury [12,13]. Interestingly no correlation was identified between neurological injury and the number or location of K-wires. Kocher et al. examined 52 patients with supracondylar humerus fractures stabilised with lateral versus bilateral K-wires and did not detect any difference in nerve injury, range of motion or return to function at the three-month follow-up [14]. Pavone et al. compared 22 patients treated with bilateral, crossed K-wires with 13 patients treated with lateral K-wires only. They found both groups to have no severe adverse effects or persisting impairments, and treatment led to good recovery [15]. No clinical difference was detected in our study, in which up to four K-wires were used, indicating that, if adequately performed, both crossed or lateral k-wire pinning methods might be safe. 

Importantly, the overall rate of iatrogenic neurovascular injury was less than 6%, in line with previously published data [4]. In contrast to the current findings, the ulnar nerve is generally considered the most vulnerable during operative management, particularly in supracondylar fractures, with an incidence of iatrogenic injury between 3–9%, increasing with fracture severity [4,9,16]. No significant correlation was detected in this study between the type of fracture surgical technique and patient age. Joiner et al. prospectively investigated the rate of iatrogenic nerve injuries in operatively treated supracondylar fractures. Rigorous examination of clinical status in compliant patients by a specialised paediatric orthopaedic surgeon demonstrated the rate of iatrogenic nerve injury to be 3% [13]. The majority of neurovascular injuries were due to initial trauma and associated with the degree of displacement [12,13]. In the current study, the distal humerus fracture subtype showed no significant effect. Various classifications systems exist for SCHF, namely Baumann, Lubinus, Felsenreich, Gartland, Holmberg and v. Laer. Gartland is the one that is primarily used in Anglo-American areas. Nevertheless, in this survey v. Laer classification was used, as it is the predominant classification in Germany [4].

The impact of time to operative intervention on the incidence of neurovascular dysfunction was not investigated in this study. There is no consensus in the literature regarding the urgency of operative intervention, a fact recently highlighted following a large survey of the members of European Paediatric Orthopaedic Society [17]. Even so, late-night treatment of supracondylar fractures has been reported to be associated with a higher risk of postoperative paraesthesia and suboptimal fixation quality [16,18,19]. However, contradicting reports exist. Farrow et al. [20] and Schmid et al. [21] suggested that postponing surgery is a safe method, and no difference in outcomes has been reported between daytime and night-time surgical treatment [22]. Accepted general practice involves fast-tracking operative treatment to avoid complications related to delayed management and subsequent soft tissue swelling. The likelihood of open reduction and associated surgical complications, such as nerve injury, compartment syndrome and infection, is minimised through timely operative management in the absence of unwieldy soft tissues [23,24]. Some have postulated that postponed acute operative management within 24 h may achieve equal or better results, as surgical accuracy in reduction and K-wire placement are responsible for the most common complications of malunion and iatrogenic nerve injury in displaced supracondylar fractures [8,24,25]. Additionally, although supracondylar fractures are the dominant subtype of fractures in the paediatric population, epicondylar fractures account for approximately 10% (lateral) and 20% (medial) of patients and are often overlooked. Fractures of the medial epicondyle are the dominant fracture subtype in patients from 13 to 15 years of age. Our study demonstrates a considerable incidence of perioperative neurovascular complication for these fracture subtypes. The importance of reliable primary clinical examination in the preoperative assessment should not be underestimated. Importantly, vascular status is difficult to assess due to the lack of compliance, oedema, haematoma and casting. Vu et al. evaluated 50 patients with injury of the brachial artery and concluded that Doppler sonography was not consistent with the severity of the intraoperative brachial artery injury [26]. However, the indication for CT angiography is still debated and should not delay fracture reduction [27]. Tomaszewski et al. reported good clinical results for children with SCHF and vascular injury at the one-year follow-up [12], which is in line with our study data. Our study demonstrates mostly excellent clinical outcomes at mid-term follow-up. Mild varus or valgus joint instability was often demonstrable but not associated with pain or an elevated QuickDash score. We concluded that minor joint instability under 10 degrees did not result in relevant functional disability. Additionally, elbow stiffness, a common consequence of elbow injury in the adult population, was not identified as a complication in the paediatric population of this study. Bernthal et al. identified elbow stiffness as a transient complication in supracondylar humerus fractures [28]. Follow-up examination of the eight patients with neurovascular dysfunction at discharge demonstrated a return of full neurovascular function in six patients, including one patient who underwent a second procedure with decompression neurolysis of the ulnar nerve. In one case of complex traumatic dissection of the brachial artery with median and ulnar nerve dysfunction, only a residual neurapraxia with paradoxical temperature sensation persisted. Paediatric patients are often considered to have high potential for recovery [15]. However, this should not be taken for granted. Tomaszewski et al. demonstrated that after an initial nerve injury associated with supracondylar humerus fracture, complete nerve recovery occurred on average 122 days after surgery [12].

Consistent with our observations, posttraumatic neurological loss of function is typically caused by simple neurapraxia and therefore does not warrant specific treatment or further diagnostic investigation within the first six weeks [9]. Patients must be followed closely, and their neurovascular status must be re-evaluated at regular intervals after surgery and before any removal of hardware [11,29].

Rose et al. reported 10 postoperative injuries of the ulna nerve in a series of 114 supracondylar humerus fractures [6]. In four patients, the nerve was explored or the medial pin was removed; the remaining six patients were treated. After nine months, nine of the 10 reported full recovery. Rose concluded that due to the risk of pinning the ulna nerve, all acutely occurring neurological lesions identified within 24 h should be operatively revised. Symptoms evolving subacutely are typically a result of simple neural irritation without structural lesion and are likely to resolve spontaneously or after removal of the implants. 

Some relevant limitations of the current investigation must be acknowledged. This study was conducted retrospectively, and clinical follow-up was inconsistent. Of 84 patients satisfying the inclusion criteria, only 34 were available for clinical follow-up. Similar high losses to follow-up have been reported in related studies [14]. In our study, the reasons given for not attending clinical follow-up included distance of the place of residence to the study centre—many patients were treated as tourists travelling interstate. A further limitation of the study was related to the occasional paucity of clinical documentation. Clinical examination and documentation in the emergency department is challenging, especially in injured children [16]. Finally, only operatively managed patients were included in this study, resulting in selection bias in the severe fractures analysed in this cohort. Conclusions drawn from this study are only specifically relevant to surgically treated distal humerus fractures in paediatric patients.

## 5. Conclusions

Paediatric distal humerus fractures are associated with a considerable incidence of primary traumatic and secondary iatrogenic neurovascular complications, which must be considered when contemplating operative intervention. Accurate and reliable pre- and postoperative examination and documentation are mandatory to detect early operative or implant-related complications in the postoperative period. With standardised operative techniques and reliable follow-up examinations, these patients can achieve excellent long-term clinical outcomes.

## Figures and Tables

**Table 1:** Operation procedures depending on the fracture type.

	CRIF (k-Wire)	ORIF (k-Wire)	ORIF (k-Wire + Screw)	ORIF (Screw)	ORIF (Double Plate)	All
n (%)	38 (45.2%)	22 (26.2%)	18 (21.4%)	5 (6.0%)	1 (1.2%)	84
Supracondylar fracture	38	17	0	0	0	54
Lateral epicondyle	0	1	6	2	0	9
Medial epicondyle	0	3	10	3	0	16
Transcondylar fracture	0	1	0	0	1	2
S42.49 (Other)	0	0	1	0	0	1
Supracondylar fracture + medial epicondyle	0	0	1	0	0	1

**Table 2:** Early postoperative complications in surgically treated paediatric distal humerus fractures.

Type of Complication (n = 84)	At Initial Examination	At Time of Discharge	At 6-Week Follow-Up
Median nerve injury	3 (3.6%)	4 (4.8%)	2
Ulnar nerve injury	2 (2.4%)	2 (2.4%)	2
Ulnar nerve injury with need of neurolysis	0	1 (1.2%)	0
Radial nerve injury	0	1 (1.2%)	0
Median and radial nerve injury	2 (2.4%)	0	0
Unspecific nerve injury (tingling paraesthesia on the finger tips)	0	1 (1.2%)	0
Brachial artery dissection and median nerve injury	1 (1.2%)	0	0
Brachial artery dissection and median + radial nerve injury	1 (1.2%)	0	0
Brachial artery dissection	1 (1.2%)	0	0
Secondary displacement	0	2 (2.4%)	0
Postoperative malalignment and osteonecrosis of the Lateral epicondyle	0	1 (1.2%)	0
Limited ROM of elbow	-	0	19 (22.5%)
- Flexion	-	0	6 (7.1%)
- Extension	-	0	4 (4.8%)
- Extension and supination of forearm	-	0	1 (1.2%)
- Flexion and extension	-	0	8 (9.5%)

**Table 3:** Neurovascular injuries and their attribution to different types of distal humerus fractures.

Type of Complication	Primary Posttraumatic	Secondary/Iatrogenic
Brachial artery dissection	3 supracondylar (all type 4 *)	0
Median nerve injury	7 - 5 supracondylar (all type 4 *) - 1 lateral epicondyle - 1 medial epicondyle	3 - 1 supracondylar (type 4 *); CRIF with K-wire - 1 lateral epicondyle; ORIF with screw - 1 S42.49 (Others); ORIF with K-wire + screw
Ulnar nerve injury	2 - 1 supracondylar (type 3 *) - 1 medial epicondyle	1 - 1 supracondylar (type 3 *); CRIF with K-wire
Radial nerve injury	3 - 2 supracondylar (both type 4 *) - 1 lateral epicondyle	1 - 1 supracondylar (type 4 *); ORIF with K-wire

* According to von Laer classification.

**Table 4:** Clinical follow-op examination at mid-term.

Clinical Score	Number of Patients	%
*Quick DASH score at follow-up*		
0	16	47.1
>0–10	15	44.1
>10–20	3	8.8
>20	0	0
*Broberg & Morrey score at follow-up*		
Excellent (95–100 points)	30	88.2
Good (80–94 points)	4	11.8
Fair (60–79 points)	0	0
Poor (<60 points)	0	0
*Mayo Elbow Performance score at follow-up*		
Excellent (90–100 points)	33	97.1
Good (75–89 points)	1	2.9
Fair (60–74 points)	0	0
Poor (<60 points)	0	0

## Data Availability

The data presented in this study are available on request from the corresponding author.
